# Defining and Predicting Patterns of Early Response in a Web-Based Intervention for Depression

**DOI:** 10.2196/jmir.7367

**Published:** 2017-06-09

**Authors:** Wolfgang Lutz, Alice Arndt, Julian Rubel, Thomas Berger, Johanna Schröder, Christina Späth, Björn Meyer, Wolfgang Greiner, Viola Gräfe, Martin Hautzinger, Kristina Fuhr, Matthias Rose, Sandra Nolte, Bernd Löwe, Fritz Hohagen, Jan Philipp Klein, Steffen Moritz

**Affiliations:** ^1^ Department of Psychology University of Trier Trier Germany; ^2^ Departmemt of Psychology University of Bern Bern Switzerland; ^3^ Department of Psychiatry and Psychotherapy University Medical Center Hamburg-Eppendorf Hamburg Germany; ^4^ Department of Psychiatry and Psychotherapy Lübeck University Lübeck Germany; ^5^ GAIA AG Hamburg Germany; ^6^ Department of Health Economics and Health Care Management Bielefeld University Bielefeld Germany; ^7^ Department of Psychology Eberhard Karls University Tuebingen Tuebingen Germany; ^8^ Department of Psychosomatic Medicine Charité University Medical Center, Berlin, Germany Berlin Germany; ^9^ Department of Psychosomatic Medicine and Psychotherapy University Medical Center Hamburg-Eppendorf Hamburg Germany

**Keywords:** patterns of early change, depression, web interventions, psychotherapy research

## Abstract

**Background:**

Web-based interventions for individuals with depressive disorders have been a recent focus of research and may be an effective adjunct to face-to-face psychotherapy or pharmacological treatment.

**Objective:**

The aim of our study was to examine the early change patterns in Web-based interventions to identify differential effects.

**Methods:**

We applied piecewise growth mixture modeling (PGMM) to identify different latent classes of early change in individuals with mild-to-moderate depression (n=409) who underwent a CBT-based web intervention for depression.

**Results:**

Overall, three latent classes were identified (N=409): Two early response classes (n=158, n=185) and one early deterioration class (n=66). Latent classes differed in terms of outcome (*P*<.001) and adherence (*P*=.03) in regard to the number of modules (number of modules with a duration of at least 10 minutes) and the number of assessments (*P*<.001), but not in regard to the overall amount of time using the system. Class membership significantly improved outcome prediction by 24.8% over patient intake characteristics (*P*<.001) and significantly added to the prediction of adherence (*P*=.04).

**Conclusions:**

These findings suggest that in Web-based interventions outcome and adherence can be predicted by patterns of early change, which can inform treatment decisions and potentially help optimize the allocation of scarce clinical resources.

## Introduction

Web-based interventions for individuals with depressive disorders have been a recent focus of research and may be an effective addition to face-to-face psychotherapy or pharmacological treatment. For example, such interventions may be appropriate for individuals who have difficulty accessing psychological treatment or do not want to utilize face-to-face treatment [1-5]. Several studies suggest that some forms of Web-based interventions may be as effective as face-to-face therapy [6], although various methodological limitations of this body of research have also been noted [7]. One limitation of Web interventions is that they are not accepted by all patients and some drop out early or do not adhere to the treatment protocol [8]. Especially in unguided Web interventions, the risk of dropout is high [9,10] and results of studies on prereatment predictors of outcome in Web interventions remain inconsistent [11]. Additionally, not all Web interventions are equal with regard to their quality or evidence base [12]. So far, investigations of Web interventions have mainly focused on treatment efficacy and short-term symptom change in comparison with treatment-as-usual control groups, in which participants were only able to access the Web intervention after a delay of several weeks or months [13,14].

Whereas a good database has been established regarding the general effectiveness of several Web-based interventions for the treatment of psychological problems, there is still a lack of research investigating the process and shape of change [15]. On the other hand, this area of research has a certain tradition in individual therapy. In recent years, interest in the investigation of early change patterns and their relation to outcome has grown. The basic idea behind this research is to use early change of the target behavior (eg, depressive symptoms) to predict treatment outcome [16,17]. Early change patterns have been shown to be associated with outcome across different diagnoses [18,19], different treatment approaches [20,21], and different measures [22].

For example, a recent study investigated early change patterns in patients with panic disorder (n=326), who underwent manualized cognitive behavioral therapy (CBT) [23]. Using growth mixture modeling (GMM), 4 latent subgroups were identified, showing clusters of change trajectories over the first 5 sessions. One of the subgroups consisted of patients whose symptoms decreased rapidly and who also showed the best outcomes (early responders). This information on early response improved treatment prediction by 16.1% over patient intake characteristics. Early change patterns also significantly predicted early dropout. Likewise, a further study focused on early change patterns during low intensity interventions [24]. This study used data from patients with anxiety disorders or depression, who accessed the Improving Access to Psychological Therapies (IAPT) service in the United Kingdom (n=511). This service comprised between 1 and 8 sessions and was often delivered via telephone. Early response, defined as reliable improvement until session 4, was predictive of clinically significant recovery after treatment termination. It was noted that attrition was highest in early sessions, so that early attempts to engage patients should be made.

This example emphasizes the importance of also studying early change patterns in low intensity and Web interventions. Whereas conclusions drawn from efficacy and effectiveness studies are limited to the average patient after treatment, knowledge about early change patterns may help to answer clinical questions. For example, such knowledge could be used to predict whether the treatment in question will work for a particular subgroup or to decide whether users should continue treatment [25,26]. Such questions have become increasingly relevant in the face of the recent implementation of stepped-care models, where patients are matched to a treatment with the option of being “stepped-up” to more intensive care [27,28]. Knowledge about predictive determinants may add to the development of empirically based rules that support clinicians in their decisions [28] and may help to prevent dropout or low adherence. In addition, investigating change patterns promotes the understanding of change processes, which is necessary for treatment development efforts [15].

Although early change patterns are important predictors of treatment outcome [23,29], to date only one study has looked at early change patterns in patients undergoing Web-based interventions. Schibbye and colleagues [30] examined change patterns during a CBT-oriented Web-based intervention, which was provided to patients with panic disorder, social phobia, or depression (n=112) by the Internet Psychiatry Clinic in Sweden. Outcome of the Web intervention was predicted by estimation of early change. The prediction was best when the rating of a disorder-specific measure at week 4 was used.

In the present study, we analyzed data from a multicenter trial testing the efficacy of a CBT-oriented web-based intervention for individuals with mild to moderate depression. Based on the existing literature on individual therapy, we predicted the existence of distinct early patient response clusters in this Web intervention. We further hypothesized that these clusters would add to the prediction of treatment outcome as well as adherence. This study also examined whether initial impairment, attitudes toward Web-based interventions, and email support predict early change patterns.

## Methods

### Participants and Treatment

This study was conducted from January 2012 to December 2013 and approved by the local ethics committee (DGPs, reference number SM 04_2012). Written informed consent was obtained, and the study was registered at ClinicalTrials.gov (identifier: NCT01636752). Several settings were used to recruit participants: (1) In- and outpatient medical and psychological clinics, (2) internet forums for depression, (3) health insurance companies, and (4) the media (eg, newspaper). Participants were directed to the study’s website. In total, 2020 participants signed up for the study and were screened for inclusion and exclusion criteria. Inclusion criteria consisted of (besides Internet access) mild-to-moderate depressive symptoms defined by scores between 5 and 14 on the Patient Health Questionnaire-9 (PHQ-9) and ages between 18 and 65 years. Participants who fulfilled these criteria were further screened by telephone using the Mini International Neuropsychiatric Interview (M.I.N.I. [31]). Also, a baseline assessment was conducted using several self-report measures (see below). If PHQ-9 scores were above 14, acute suicidality was determined or a lifetime diagnosis of bipolar disorder or schizophrenia was identified in the interview [32], participants were excluded from the study, and professional help was suggested to them. Included participants (n=1013) were then randomized into either an intervention group (IG), in which a CBT-oriented Web-based intervention (Deprexis) was delivered in addition to care as usual (IG; n=509), or into a control group (CG), which solely consisted of care as usual (CG; n=504). During the study, participants in the care as usual group did not receive any Web intervention. The use of other interventions initiated by the participants in the care as usual group was measured during the course of the study. At posttreatment, participants reported having utilized the following treatments during the course of the study: medication, treatment by a psychotherapist, treatment at an outpatient clinic, and treatment at an inpatient clinic. There were no significant differences between participants in the IG and the CG regarding the use of medication (*P*=.54), treatment by a psychotherapist (*P*=.38), treatment at outpatient clinics (*P*=.68), and treatment at inpatient clinics (*P*=.29).

As incentive, all participants were entered into a lottery for 12 iPods after the last assessment. Furthermore, participants in the CG received access to the Web intervention 1 year after baseline assessments.

In addition to the pre- and post-treatment PHQ-9 assessments, which all participants filled out, participants in the IG filled out PHQ-9 assessments every 2 weeks during the course of the study. Furthermore, participants in the IG who had mild symptoms of depression (PHQ-9 scores between 5-9), received the Web-based intervention without any guidance, whereas participants who had moderate depressive symptoms (PHQ scores between 10-14) received the same Web intervention in combination with weekly email support [33,34]. Studies have shown that unguided Web interventions are also effective in the treatment of depression (eg, [35]). However, considering safety and efficacy, more intensive support seemed appropriate for patients with moderate depression.

After randomization into the IG, participants had to register on the study’s website and were then able to use the Web-based intervention (Deprexis) for a period of 12 weeks. It is based on a cognitive-behavioral approach and consists of 10 modules that are presented in the form of a dialogue or “chat.” The modules contain classic CBT elements such as behavioral activation, but also broader therapeutic elements such as mindfulness, emotion-focused interventions, and interpersonal skills. Information as well as advice on the application of the relevant concepts in daily life were combined in the modules, which included text, illustration, and audio. This content was presented in dialogue form, where the user was asked to select one of several response options to the program’s explanations. In total, several published randomized controlled trials have provided evidence in support of the program’s efficacy, typically with small-to-medium effect sizes [35,32,3].

**Figure 1:**
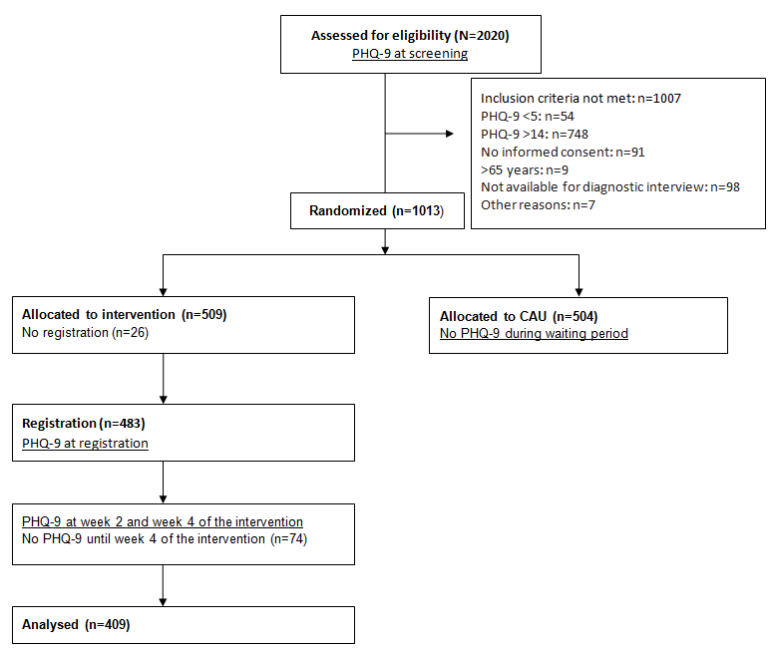
Flowchart of participants.

In this study, our main interest was to examine the change patterns of participants who received the intervention. Therefore, we focused on participants for whom not only pre- and post-assessments were available, but also several PHQ-9 assessments from during participation. This condition was fulfilled by participants in the IG (PHQ-9 every 2 weeks), but not by participants in the CG (pre and post PHQ-9 assessments only). In total, 483 of the 509 participants randomized into the IG registered on the study’s website. A total of 409 participants filled out at least one PHQ-9 during the first 4 weeks of the intervention (assessment at week 2 or assessment at week 4, see flowchart in Figure 1) and were therefore included in our study sample. Participants without any assessment during the intervention were excluded, as no meaningful course of change could be modeled for those cases. Participants (n=409) included in the study, and participants who did not register (n=26) or did not complete any assessment during the intervention (n=74) did not differ with regard to age (*F*_2,506_=1.18, *P*=.31), gender (χ²_2_=2.2_,_*P*=.34), or initial impairment (PHQ-9 at screening, *F*_2,506_=1.47, *P*=.23).

On average, 2 weeks passed between screening and registration (standard deviation, SD=1.36). The first assessment took place 2 weeks after registration. Most participants were recruited by online forums (n=82), health insurance companies (n=134), or learned of the study by other means, commonly by news in media (n=236). Other participants learned of the study while in treatment (n=57). Attrition (n=100) did not differ between the recruiting options described previously (χ²_12_=18.0, *P*=.12).

On average, participants were 43.16 years old (SD=11.10, range=18-65) and approximately 70% of participants (287/409) were women. Close to 50% of participants had a high-school diploma qualifying for university entrance (204/409). Most participants (n=264) suffered from moderate depression and therefore received the Web intervention as well as additional weekly brief support via email. In this study, early response was estimated based on PHQ-9 score changes between Web-based registration and week 4 of the intervention. In addition to the PHQ-9, other impairment measures and participant attitudes were assessed at screening and posttreatment (see questionnaires below).

### Measures

#### Diagnostic Interview

Diagnoses were made using the M.I.N.I. [31], and clinician-rated severity of depression was assessed with the 24-item version of the Hamilton Depression Rating Scale (HDRS-24). The M.I.N.I. is a short structured diagnostic interview for Diagnostic and Statistical Manual of Mental Disorders, 4th Edition (DSM-IV) and International Classification of Diseases, Tenth Edition (ICD-10) disorders that has been translated into multiple languages. In several studies, it has shown good interrater reliability (eg, [36]). Acute suicidality was assessed based on current suicidal ideation and past suicide attempts. In this study, trained raters (postgraduate students) conducted the interviews via telephone. Before they were permitted to rate trial participants, raters were trained to conduct the interview either face-to-face or via telephone modules and had to demonstrate adequate interrater reliability on an audiotaped interview.

#### Patient Health Questionnaire-9 (PHQ-9)

The PHQ-9 consists of 9 items that reflect the criteria of depression in DSM-IV [37]. Answers are provided on a 4-point Likert scale (0-“not at all” and 3-“nearly every day”). Thus, total scores range from 0-27, with scores between 5 and 9 indicating mild depression and scores between 10 and 14 indicating moderate depression. The instrument has a good test-retest reliability (r_tt_=.84) and internal consistency (Cronbach alpha=.86-.89; [37]). To operationalize reliable improvement, the reliable change index (RCI), which reflects the pre- post treatment difference ΔRC large enough to not be attributable to measurement error, was calculated following Jacobson and Truax (see [38]): where r is the reliability of the PHQ-9 (*r*=.86) and SD the standard deviation of the PHQ-9 intake score (SD=2.37). The RCI score for the PHQ-9 was 2.46 total points.

#### Short-Form Health Survey-12 (SF-12)

The SF-12 assesses limitations in role functioning with 12 items. It consists of two subscales measuring physical health (SF-12_Physical Health Scale_) and mental health (SF-12_Mental Health Scale_) [39]. Presence and severity of different impairments over the last 4 weeks are rated. Subscale scores can vary between 0-100, with higher scores indicating less impairment. Reliability is good with a Cronbach alpha of .76 [40] and test-retest correlations of r_tt_=.76 for the physical component and r_tt_=.89 for the mental component [39].

#### Questionnaire for the Evaluation of Psychotherapeutic Progress-2 (FEP-2)

The FEP-2 comprises 40 items and measures 4 dimensions of therapeutic progress and outcome (well-being, symptom distress, incongruence, and interpersonal problems) [41]. Answers are provided on a 5-point Likert scale (1-“never” and 5-“very often”) with higher scores indicating higher impairment. Reliability is high for the global scale (Cronbach alpha=.96; Retest between r_tt_=.69-.77) and sensitivity to change has been demonstrated [41].

#### Attitudes Toward Psychological Online Interventions (APOI) Questionnaire

The attitudes toward psychological online interventions (APOI) [42] measures attitudes toward online-based interventions with 16 items. The following subscales are assessed: (1) Confidence in Effectiveness, (2) Skepticism and Perception of Risks, (3) Technologization Threat, and (4) Anonymity Benefits. Answers are provided on a 5-point Likert scale (1-“I disagree entirely” and 5-“I agree entirely”) and subscale scores range from 4-20. Higher values on the APOI total score indicate a more positive attitude toward psychological online interventions (POI). Reliability is good with a Cronbach alpha of .77.

#### Adherence

Adherence to the intervention was defined as the extent to which participants used the intervention. A number of modules were calculated by summing up all modules that were accessed for at least ten minutes. Usage time was defined as the number of hours participants spent using the Web intervention. At screening, at registration, every 2 weeks during the 12-week Web intervention period, and after the intervention, participants were asked to fill out the PHQ-9. The number of completed PHQ-9 assessments after week 4 of the intervention was used as an additional indicator of adherence.

#### Data Analytic Strategy

Patterns of early change in depressive symptoms, measured by the PHQ-9 over the first 4 weeks of the Web intervention, were identified using piecewise growth mixture modeling (PGMM) [43]. GMMs are considered a conservative method of identifying early change patterns in comparison with rational definitions such as reliable or clinical significant change [44]. Individual variance of intercepts (intake scores) and slopes (change) are captured in terms of a latent class variable that is added to the growth model [43], which allows the identification of subpopulations of participants with similar growth curves. In contrast to conventional growth models, which assume that there is only one underlying population with a single change pattern, GMMs allow the investigation of an a priori unknown number of latent subpopulations, which can differ with regard to intercepts and slopes (in the case of a linear model) as well as class specific variations around these parameters. In GMM, cases with a missing value in the PHQ-9 over the first 4 weeks were not excluded, but rather all available data was used to estimate growth curves within clusters.

In this study, we applied a PGMM, modeling the change pattern as 2 distinct phases (phase 1: time between screening and registration; phase 2: time between registration and assessment at week 4 of the intervention). Therefore, we used a model with 3 latent growth factors: an intercept indicating initial impairment and 2 slopes (one for each phase of change). To model change before the intervention (phase 1), the first slope loadings, which represent change in phase 1, were fixed to 0 at screening and to 1 at registration and later assessments. To model change during the first 4 weeks of the intervention (phase 2), the second slope loadings, which represent change in phase 2, were fixed to 0 at screening and registration and for the following 2 assessments, the log-linear transformation (base 10) of 2 and 3 were used respectively. According to the Bayesian information criterion (BIC; [45]), the log-linear transformation of factor loadings for the second slope improved the model fit compared with the linear transformation and was therefore used in subsequent analyses (linear: 6826.98, log-linear: 6820.20).

In order to model early response while taking potential spontaneous remission into account, we implemented one categorical latent class factor based on the 3 growth parameters (intercept, first slope, and second slope). Several fit criteria had been discussed to determine the optimal number of latent trajectory classes. In this study, we applied the BIC and the bootstrapped likelihood ratio test (BLRT) as proposed by Nylund et al [46] to determine the optimal number of latent trajectory classes. Thus, the model determination process was 2-fold. To identify the model with the lowest BIC value, the estimation procedure started with a 1-class solution and then one more class was added in each subsequent run. As mixture models are sensitive to class overextraction, in the second step an additional criterion (BLRT) was used to balance against this potential bias. Once the BIC value no longer decreased from a model with k classes to a model with k+1 classes, this solution was then tested against a solution with k-1 classes using the BLRT. If the BLRT revealed a significant *P* value (*P*<.05), the model was chosen as the best solution. If, however, the BLRT was not significant, the model was rejected and the solution with one class less (k−1) was tested against a model with two classes less (k−2). This procedure was repeated until the BLRT resulted in a significant *P* value.

In the final analysis, we fixed the variances around the class-specific slopes to zero in both phases, whereas intercept variances were freely estimated but constrained to be constant between classes. Therefore, heterogeneity in change had to be captured by the difference in mean slopes of different latent classes completely. Thus, in line with our main interest, we forced the estimation procedure to be more sensitive to patterns of change over time rather than to differences of initial levels of impairment. This approach can be seen as a hybrid of models in which all parameters’ variances are fixed to zero (latent class growth models) and in which the free estimation of all parameters is allowed (for similar approaches, also see [22,47]).

As the purpose of this study was to evaluate the impact of early change on overall treatment response, we examined the effect of early change patterns on change from pre- to post-treatment in terms of effect sizes as well as reliable change. To evaluate change on the PHQ-9, within-group effect sizes were calculated by subtracting the PHQ-9 score at post from PHQ-9 at screening and dividing the result by the SD of the PHQ-9 score at screening. As described previously, reliable change criteria were applied to the change scores to classify patients into 3 groups: reliably improved (pre to post improvement larger than the RCI of the PHQ-9, which equals 2.46 total points), reliably deteriorated (pre to post deterioration larger than the RCI), and not reliably changed (pre to post change remained under the RCI).

Subsequently, the identified latent change patterns were used to predict outcome and adherence, while controlling for initial impairment (PHQ-9 at screening, HRSD-24 at screening), patient characteristics (FEP-2, SF-12_Physical Health Status_ and SF-12_Mental Health Status_), and attitudes toward the online intervention (APOI_total_) in stepwise regression analysis. Finally, we examined whether initial impairment, patient characteristics, and attitudes toward the online intervention predicted early change patterns using analysis of variances (ANOVAs) and multinomial regression analysis.

## Results

### Patterns of Early Change

Following the 2-old model determination process, the 3-class solution showed the best model fit, as suggested by the BIC and BLRT (see Table 1). As a result, the 3-class solution was used for further analyses.

**Table 1:** Information criteria, entropy, and *P* values in a bootstrapped likelihood ratio test for up to 4 latent classes in a 2-piece model.

# Classes	BIC^a^	SABIC^b^	AIC^c^	Entropy	BLRT^d^*P* value
1	6855.68	6830.30	6823.57		
2	6782.91	6744.84	6734.75	0.74	<.001
3	6773.41	6722.64	6709.19	0.65	<.001
4	6777.33	6713.87	6697.06	0.66	<.001

^a^BIC: Bayesian information criterion.

^b^SABIC: sample size adjusted BIC.

^c^AIC: Akaike information criterion.

^d^BLRT: bootstrapped likelihood ratio test.

**Figure 2:**
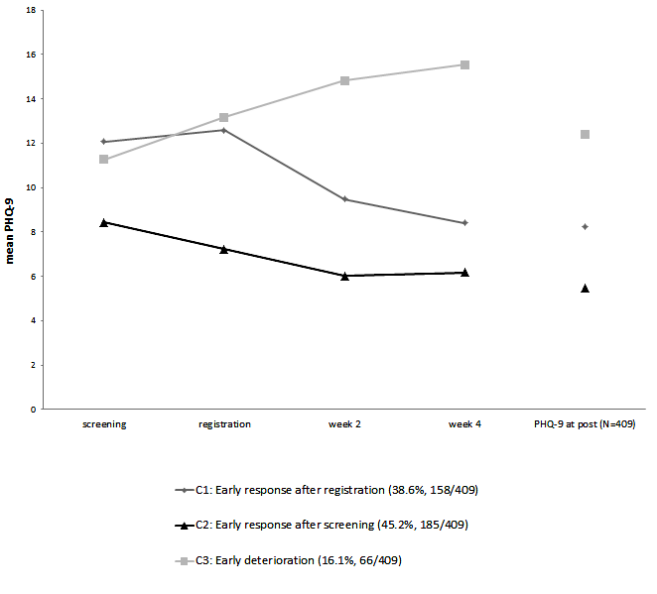
Mean latent growth curves for piecewise growth mixture modeling (PGMM) solution with 3 latent classes within the first 4 weeks and observed mean scores (Patient Health Questionnaire-9, PHQ-9) in the respective classes after the Web intervention.

Graphical inspection revealed 2 early response groups and 1 early deterioration group. As shown in Figure 2, patients in classes 1 and 3 were characterized by higher PHQ-9 scores at screening that were above the cut-off score of 9 for clinical samples (C1: mean=12.08, C3: mean=11.27). Class 2 started treatment with lower (mild) depressive symptom severity (C2: mean=8.44).

The first subgroup labeled “early response after registration” (C1: 38.6%, 158/409) showed rapid early decrease in depressive symptom severity after registration. The early change effect size (between screening and week 4) in this group was *d*=1.35, reflecting rapid improvement. In the second subgroup which was labeled “early response after screening” (C2: 45.2%, 158/409), depressive symptoms decreased significantly not only during phase 2, but already during phase 1. The early response effect size within this latent class was large (*d*=0.98). In contrast to these 2 groups, a third subgroup of participants (C3: 16%, 66/409) showed a significant increase of depressive symptoms from screening to registration and from registration to assessment at week 4. This was the only class with a negative early change effect size (*d*=−1.78) and was therefore labeled “early deterioration.”

As some participants received additional treatment during the Web intervention and some were provided with email support, we compared these variables between classes to control for differential influences. The number of patients who were in therapy at the beginning of the Web intervention did not differ across classes (χ²_2_=4.4, *P=*.11), and there was no difference between classes in regard to reported change in additional treatment status at the end of the Web intervention (χ²_10_=10.6, *P*=.39). Also, at the beginning of treatment, there was no difference between classes with regard to the number of patients receiving medication (χ²_2_=1.4, *P*=.50). At the end of treatment, classes did not differ with regard to number of patients reporting change in medication (χ²_2_=0.9, *P*=.64) or use of medication (χ²_2=_ 4.1, *P*=.13). Also there was no difference regarding use of psychotherapy (χ²_2_=0.2, *P*=.92). Only 9 patients reported being treated in outpatient clinics, and only 5 reported being treated in inpatient clinics, so no meaningful difference between classes could be established.

Furthermore, the number of patients receiving email support during the Web intervention differed significantly between classes (*P*<.001). Whereas almost all participants in C1 (96.2%, 152/158) and C3 (83%, 55/66) received email support, only 31% (57/185) of participants in C2 exceeded the cut-off of 10 on the PHQ-9 at screening and thus received email support. Therefore, email support was included as a predictor variable in the following analyses.

### Patterns of Early Change and Treatment Outcome

Table 2 shows the relative frequency of reliable improvement and pre-post effect sizes on the PHQ-9 depending on class membership. The relationship between reliable improvement of depressive symptoms and class membership was analyzed with a chi-square test, which revealed a significant association (χ²_9_ =74.8, *P*<.001).

As can be seen in Table 2, 62% (99/158) of participants in C1 (early response after registration) showed reliable change (standardized residual=1.5) and, on average, participants in this group showed the largest pre-post effect size (*d*=1.63). Only 3% (5/158) showed a reliable negative development at the end of treatment.

In C2 (early response after screening) the rate of reliable improvement (56%, 104/185) and the pre-post effect size (*d*=1.25) were slightly lower than in C1. The rate of reliable deterioration was 7% (12/185) for this class.

In C3 (early deterioration) only 27% (18/66; standardized residual=−3.00) of participants showed reliable improvement, yet in 39% of cases (26/66; standardized residual=7.2), participants in this class showed reliable deterioration. This was also the only class with a negative pre-post effect size (*d*=−0.47). All 3 classes had similar rates of lacking reliable change (C1: 34.2%, 54/158; C2: 37.2%, 69/185; C3: 33%, 22/66).

To identify potential relevant predictors beyond impairment at screening (PHQ-9, HRSD-24), we first correlated patient characteristics (FEP-2, SF-12_Physical Health Status_ and SF-12_Mental Health Status_) and attitudes toward online interventions (APOI_total_) with PHQ-9 postscores. Only initial attitudes toward online interventions (*r*=−.12, *P*=.01) were significantly correlated with outcome.

Subsequently, the estimation of the additional predictive power of early change patterns beyond variables at screening was conducted via a stepwise regression analysis. Impairment at screening (PHQ-9) was added into the model first, followed by HRSD-24 and APOI_total_. The dummy coded class membership variables were added to the model in the last stage of the analysis (see Table 3). The inclusion of the PHQ-9 score at screening explained 7.6% of the variance of PHQ-9 at post (*P*<.001). Participants with higher scores at the beginning of treatment tended to end with higher scores after treatment. HRSD-24 was also included and significantly increased the amount of explained variance by 3.4% (*P*<.001). Similarly, participants with higher impairment scores tended to end with higher scores. The addition of APOI_total_ significantly increased the amount of explained variance by a further 1.5% (*P*=.008) resulting in a total of 12.5% of explained variance. A higher score at intake, indicating a more positive attitude toward the intervention, was significantly associated with lower PHQ-9 scores after treatment (see Table 3). Email support was not significant and therefore excluded in the next step (*t*_407_=−0.05, *P*=.96).

**Table 2:** Relative frequencies of reliable improvement of the Patient Health Questionnaire-9 (PHQ-9), pre-post change on the PHQ-9 (effect sizes) and adherence by patient group of early change.

Sample		Outcome		Adherence		
	n	Reliable improvement on PHQ-9^a^ n (%)	Pre-post ES^b^ on PHQ-9 (*d*) (95% CI)	Usage time (in hours) mean (SD^c^)	Number of modules of the Web intervention mean (SD)	Number of assessments^d^ mean (SD)
All patients	409	221 (54)	1.12 (0.94-1.30)	7.89 (4.81)	9.10 (4.38)	2.52 (1.25)
Class 1^e^	158	99 (62)	1.63 (1.39-1.86)	8.36 (4.08)	9.84 (3.90)	2.80 (1.16)
Class 2^f^	185	104 (56)	1.25 (1.04-1.47)	7.32 (4.69)	8.64 (4.52)	2.37 (1.25)
Class 3^g^	66	18 (27)	−0.47 (−1.05 to 0.12)	8.33 (6.39)	8.65 (4.85)	2.27 (1.36)
*P* value		<.001^h^	<.001^i^	.10^i^	.03^i^	<.001^i^

^a^PHQ-9: Patient Health Questionnaire-9.

^b^ES: effect size.

^c^SD: standard deviation.

^d^Number of assessments: Number of PHQ-9s after week 4.

^e^Class 1: Early response after registration.

^f^Class 2: Early response after screening.

^g^Class 3: Early deterioration.

^h^χ^2^ tests were performed, testing the association between class membership and categorized treatment outcome.

^i^1-way analysis of variances (ANOVAs) were performed, testing the association between class membership and mean d for pre-post change, usage time, number of modules of the Web intervention, and number of assessments.

Adding the dummy coded variables for class membership resulted in a further increase of 21.5% explained variance of treatment outcome (*P* ≤.001). Thus, in total, 34% of variability of PHQ-9 change during the course of treatment was able to be explained by the model that contained initial PHQ-9, HRSD-24, and APOI_total_ scores, as well as early change patterns (see Table 3).

### Patterns of Early Change and Adherence

Adherence assessed via the number of modules and number of assessments (number of completed PHQ-9s) was significantly associated with class membership (see Table 2). A 1-way ANOVA revealed significant associations between mean number of modules of the Web intervention and class membership (*F*_2,406_=3.65, *P*=.03). A post hoc test using Bonferroni correction showed that participants in C1 had accessed significantly more modules of the Web intervention than participants in C2 (*d*=0.28; pooled SDs between clusters were used to calculate between group effect-sizes). Concerning the number of assessments (*F*_3,405_=6.91, *P*=.001), similar results were found using Bonferroni corrected *P* values: Participants in C1 filled out more assessments than participants in C2 (*d*=0.36) and C3 (*d*=0.44). There was no significant difference between the subgroups regarding usage time (*F*_2,406_=2.32, *P*=.10).

For adherence measured by the number of modules of the Web intervention used and the number of assessments completed, the predictive power of early change patterns was examined using stepwise regression analysis (see Table 3). In the first step of the analysis, impairment at screening (PHQ-9) was included in the equation. It was significantly associated with number of assessments (*F*_1,406_=15.21, *P*<.001) and explained 3.6% of variance. HRSD-24 at screening was excluded (*t*_407_=−0.44, *P*=.66) and neither attitudes toward Web interventions (*t*_407_=−0.03, *P*=.98) nor email support (*t*_407_=0.83, *P*=.41) enhanced predictability. In the second step, class membership was entered in the model. The addition of class membership explained an additional 1.5% of variance of number of assessments (*P*=.04). Thus, a total of 5.1% of variability of number of assessments was able to be explained by the final model, which included PHQ-9 at screening and class membership.

**Table 3:** Stepwise multiple regression analyses predicting outcome and adherence by patient characteristics, email-support, and patterns of early change.

Steps	Predictors^a^	Outcome			Adherence					
		PHQ-9^b^ at post			No. of assessments^c^			No. of modules in intervention		
		ΔR^2^	Beta	*P*	ΔR^2^	Beta	*P*	ΔR^2^	Beta	*P*
Step 1		0.125		.008	0.036		<.001	0.024		.002
	PHQ-9		.216	<.001		.190	<.001		.155	.002
	HRSD-24^d^		.199	<.001		−0.043	.41		.051	.33
	APOI^e^		−0.124	.008		.005	.92		.082	.10
	Support_Email_		−0.004	.96		.066	.45		.002	.98
Step 2		0.215			0.015		.04	0.006		.31
	PHQ-9		−0.040	.50		.192	.007		.146	.04
	HRSD-24		.045	.32		−0.024	.66		.068	.22
	APOI		−0.129	.002		.001	.98		.080	.11
	Support_Email_		−0.068	.36		.073	.41		.003	.97
	C2^f^-dummy		−0.346	<.001		−0.030	.69		−0.033	.66
	C3^f^-dummy		.342	<.001		−0.132	.01		−0.082	.13
Total R^2^		.34			.05			.03		
N		409			409			409		

^a^Predictors included Patient Health Questionnaire-9 (PHQ-9) at screening, 24-item Hamilton Rating Scale for Depression (HRSD-24) at screening, attitudes toward Web-based intervention, email support, and early change patterns.

^b^PHQ-9: Patient Health Questionnaire-9.

^c^No. of assessments = No. of PHQ-9 assessments after week 4.

^d^HRSD-24: 24-item Hamilton Rating Scare for Depression.

^e^APOI: attitudes toward psychological online interventions.

^f^C2 and C3 are dummy coded class membership variables with Class 1: early response after registration, used as reference class.

### Prediction of Early Change Based on Patient Intake Characteristics

Next, we investigated the relationships between class membership, initial impairment, participants’ intake characteristics and attitudes toward Web interventions via separate ANOVAs (APOI_total_, SF-12_Physical Health Scale_, SF-12_Mental Health Scale_,_and_ FEP-2). Using Bonferroni corrected *P* values, baseline scores on the FEP-2, SF-12_Physical Health Scale_, and SF-12_Mental Health Scale_ showed significant relationships with class membership.

With regard to the SF-12_Physical Health Scale_, C3 participants showed significantly lower values than C1 (*d*=0.40) and C2 (*d*=0.43) participants, indicating a higher level of physical impairment in C3 participants. On the SF-12_Mental Health Scale_, C2 participants reached significantly higher values than C1 (*d*=0.76) and C3 (*d*=0.49) participants, indicating that participants in C2 were less mentally impaired than participants in C1 and C3. Impairment measured by the FEP-2 differed significantly between C2 and C1 (*d*=0.42) as well as C2 between and C3 (*d*=0.44), with C2 showing the lowest values, indicating the lowest level of impairment.

When adding these significant variables and email support to multinomial logistic regressions, depressive symptoms measured by the PHQ-9 (χ²_2_=75.4; *P*<.001) and HRSD-24 (χ²_2_=34.8; *P*<.001) as well as physical health (SF-12_Physical Health Scale_; χ²_2_=6.6; *P*=.04) demonstrated specific predictive power for class membership. Results of multinomial logistic regression analyses with patient characteristics as predictors of class membership are presented in Table 4.

PHQ-9 intake scores significantly discriminated between classes. Higher scores were associated with a lower probability of belonging to C3 or C2 compared with C1 and a higher probability of belonging to C3 compared with C2. In addition, HRSD-24 intake scores also discriminated between classes with higher scores associated with a lower probability of belonging to C2 compared with C1 and a higher probability of belonging to C3 compared with C2. Higher SF-12_Physical Health Scale_ intake scores, indicating lower impairment, were associated with a lower probability of membership in C3 compared with C1.

**Table 4:** Prediction of class membership by patient intake characteristics via multinomial logistic regression analyses. R^2^=.51 (Cox &amp; Snell) and 0.588 (Nagelkerke). Model χ^2^_8_=284.3. For each comparison, the class mentioned first is used as the reference class in the multinomial logistic regression.

Variables			95% CI for odds ratio		
	regression coefficient B (standard error)	*P*	Lower	Odds ratio	Upper
Class 1^a^ versus class 2^b^					
Intercept	14.84 (2.96)	<.001			
PHQ-9^c^	−1.31 (0.19)	<.001	0.18	0.27	0.39
HRSD-24^d^	−0.12 (0.03)	<.001	0.84	0.89	0.94
FEP-2^e^	0.19 (0.67)	.78	0.32	1.20	4.46
SF-12^f^_Physical Health_	−0.01 (0.02)	.73	0.95	0.99	1.03
SF-12_Mental Health_	0.02 (0.03)	.44	0.97	1.02	1.08
Email support	0.07 (0.65)	.91	0.30	1.08	3.83
Class 1 versus class 3^g^					
Intercept	3.65 (2.72)	.18			
PHQ-9	−0.26 (0.13)	.04	0.60	0.77	0.98
HRSD-24	0.04 (0.02)	.08	0.99	1.04	1.09
FEP-2	0.37 (0.60)	.54	0.45	1.45	4.68
SF-12_Physical Health_	−0.05 (0.02)	.01	0.92	0.96	0.99
SF-12_Mental Health_	0.01 (0.03)	.70	0.96	1.01	1.06
Email support	−0.77 (0.68)	.26	0.12	0.47	1.75
Class 2 versus class 3					
Intercept	−11.19 (3.12)	<.001			
PHQ-9	1.05 (0.20)	<.001	2.55	2.87	4.21
HRSD-24	0.16 (0.03)	<.001	1.07	1.18	1.25
FEP-2	0.19 (0.73)	.80	0.29	1.21	5.07
SF-12_Physical Health_	−0.04 (0.02)	.07	0.92	0.96	1.00
SF-12_Mental Health_	−0.01 (0.03)	.70	0.93	0.99	1.05
Email support	−0.84 (0.65)	.20	0.12	0.43	1.56

^a^Class 1= early response after registration.

^b^Class 2= early response after screening.

^c^PHQ-9: Patient Health Questionnaire.

^d^HRSD-24 = 24-item Hamilton Rating Scale for Depression.

^e^FEP-2: Questionnaire for the Evaluation of Psychotherapeutic Progress-2.

^f^SF-12: 12-item short form health survey.

^g^Class 3: early deterioration.

## Discussion

### Principal Findings

This study examined patterns of early change during the first 4 weeks of a 12-week CBT-oriented Web-based intervention for depression by applying a PGMM analysis. We were able to identify 3 early change patterns: The first was characterized by early improvement after screening, the second by early improvement after registration, and the third by early deterioration. Furthermore, latent classes differed with regard to outcome and adherence measured by the number of assessments (number of completed PHQ-9s) and number of modules used (for a duration of at least ten minutes), but not with regard to the overall amount of time spent using the system. Class membership improved outcome prediction by 21.5% over impairment at intake (PHQ-9 at screening, HRSD-24) and attitudes toward online interventions (APOI). In addition, initial impairment on the PHQ-9 and class membership significantly predicted the number of assessments. Furthermore, group membership of patients was significantly predicted by initial impairment on the PHQ-9 and HRSD-24 as well as by impairment on the SF-12 scale physical health.

The early response and deterioration patterns identified in this study of a CBT-oriented Web-based intervention have also been found in studies of individual face-to-face therapy [23]. In this study, a more differentiated investigation of early response was made possible by including the phase from screening to registration in the analysis. The identification of a subgroup that improved before treatment started may be indicative of a regression to the mean or “spontaneous remission” effect for some patients. Yet spontaneous remission may only explain part of the effect in this group. The decision to start treatment and the knowledge of being screened and accepted for the Web intervention may have already created a positive effect by inducing hope and positive treatment expectations, therefore leading to continuous positive changes in outcome, reaching to the end of treatment. Interestingly, participants in this class had significantly less email support than the other 2 classes. Clearly, more studies, which consider the pretreatment phase in the study of early change patterns, are necessary.

In contrast, early response after registration may correspond to a response pattern, which has recently been described as the pliant response pattern [48]. For this patient group, the impact of the specific treatment is essential: response to treatment is excellent, if the treatment provided is excellent and poor and if the treatment provided is poor. In line with the results of previous studies [24,23], participants with early positive change were likely to be improved (reliably) at the end of the treatment and, on average, showed a higher mean effect size than other participants.

In our study, the rate of participants showing early deterioration (16.1%, 66/409) was somewhat higher than in other studies (4.6%, [23]; 2.4%, [43]), yet more studies are required before conclusions regarding the risk of deterioration during Web interventions can be drawn. Participants who deteriorate early may be facing crisis and be in need of more immediate help than can be provided by an Web intervention. They may especially benefit from treatment selection and the combination of face-to-face and Web interventions [49]. In any case, email support was not lower in this group than in the early response after registration group.

One possible explanation of the mixed findings regarding the frequency of early negative response patterns could be the varying settings, with different early response rates in face-to-face, medication, and Web interventions. However, further studies must also investigate the influence of varying (outcome?) instruments and definitions of early response within this area of investigation [23].

Although email support did not predict outcome, initial PHQ-9 and HRSD-24 scores as well as attitudes toward Web interventions remained significant predictors of outcome after controlling for class membership. The finding of an association between attitudes toward Web interventions and outcome fits well with findings concerning the contribution of treatment expectation to treatment outcome [50]. In Web interventions, one of the first aims should therefore be to promote positive attitudes and motivation with regard to the intervention, making improvement more likely, while preventing dropout.

With regard to adherence, it could be shown that participants with early symptom deterioration completed fewer modules of the intervention and fewer assessments than the early response after registration group. However, class membership predicted number of assessments only. Participants who experience improvement may feel more inclined to track their progress and to make maximum use of the limited time available (12 weeks), whereas participants who show less early improvement may be discouraged from using the intervention more intensively. While early response is often associated with shorter treatment length [51], it has also already been reported that in time-limited treatment protocols, early response participants tend to complete the protocol and are less likely to drop out of treatment [23].

Somewhat surprisingly, there was no difference between early response groups with regard to usage time. A possible explanation could be that the Web intervention was tailored to patients, resulting in individual patients taking different paths within the Web intervention. These paths varied in length, presenting participants with critical problems with more content and longer paths, which took more time.

Physical health was associated with a higher probability of belonging to the early response after registration group compared with the early deterioration group, indicating that physical health may be an important factor not only in face-to-face treatment but also in Web interventions. In addition, Web interventions may be needed, which take poor physical health into account, for example, by providing psychoeducation and/or special coping strategies for patients with symptoms of pain. This could increase adherence by addressing a possibly important concern of some participants who may otherwise feel like the intervention is not adequately targeting their problems [52]. Furthermore, poor physical health may decrease motivation and increase negative expectations such as “nothing is going to change” or “I can’t do this” leading to dropout or lack of improvement. In this case, the initiation of motivation and hope may be especially crucial. Similar to face-to-face settings, early change patterns during Web interventions may have important implications for treatment selection, the continuation and adaptation of treatment, as well as the development of new Web or blended interventions. Early response monitoring may support the decision-making process with regard to the addition of special content (eg, coping with physical impairment and enhancing positive treatment expectations) or the necessity of higher intensity treatments. Furthermore, physical health and attitudes toward Web interventions may be important factors that influence early response or early deterioration and may be useful indicators when deciding whether a specific Web intervention should be applied. Although some interventions target multiple problems [53], it is still unclear whether such interventions can raise the early positive response rate. Also, given that the participants in the early response after screening and early response after registration groups showed improvement, it may be that varying factors contribute to early response.

### Conclusions

Clearly, hope and positive expectations have an impact on early response; however, we don’t yet know much about specific personal characteristics such as self-efficacy. It would be interesting to investigate whether participants that improve or show an early positive change differ with regard to self-efficacy and whether high or low self-efficacy influences outcome in the long-term.

To summarize, more research is still necessary to understand which factors contribute to early response, which factors are indicate risk of early deterioration or dropout, and how clinicians or developers of Web interventions can best adapt interventions, particularly in routine care settings [53,54].

In summary, identifying patterns of early change can have implications for treatment outcome and treatment completion rates. Session-by-session monitoring and feedback of this information may increase awareness of these early change patterns and be applied as part of a stepped-care treatment approach [49].

### Limitations

The following limitations of this study should be considered when interpreting the results. Unfortunately, number of modules of the Web intervention and usage time could not be assessed on a weekly basis, limiting what can be said about the progress of adherence in relation to the progress of symptoms. Also, due to economic considerations, the PHQ-9 was used as the sole outcome measure over the course of treatment. In future studies, a broader range of outcome measures (eg, an anxiety measure) and usage variables could be regularly monitored, improving the estimation and investigation of outcome and adherence. In future studies, additional predictors of adherence should also to be studied.

In addition, only participants with at least one assessment during the intervention were included in the analyses. We addressed this issue by testing for differences between included and excluded participants. Although we did not find any differences, these results should not be generalized to participants who, for whichever reason, did not complete any assessments during the first weeks of treatment.

In addition, it must be mentioned that the application of GMM and the associated selection of an optimal number of groups is not without disadvantages [55,56]. One disadvantage is the possibility of specification errors, which can result in the overextraction of trajectory classes through GMM [57]. For this reason, after examining 2 common fit indices (BIC and BLRT), we decided to take the 3-class model into account only. When interpreting early change patterns extracted using GMM, it should not be forgotten that the result is a simplification of a more complex reality, which warrants caution [55]. GMM remains just one possibility to identify early change patterns and other identification possibilities should be considered.

Despite these limitations, this study underlines the potential of early change patterns as predictors of treatment outcome as well as adherence, which, in the future, may guide treatment decisions regarding the content and continuation of Web interventions.

## Abbreviations

AIC: Akaike information criterion
ANOVA: analysis of variances
APOI: attitudes toward psychological online interventions
BIC: Bayesian information criterion
BLRT: bootstrapped likelihood ratio test
C1: Class 1: Early Response after registration
C2: Class 2: Early Response after screening
C3: Class 3: Early deterioration
CBT: cognitive behavioral therapy
CG: control group
DSM-IV: Diagnostic and Statistical Manual of Mental Disorders, 4th Edition
ES: effect size
FEP-2: Questionnaire for the Evaluation of Psychotherapeutic Progress-2
GMM: growth mixture modeling
HDRS-24: Hamilton Depression Rating Scale
IAPT: Improving Access to Psychological Therapies
ICD-10: International Classification of Diseases, Tenth Edition
IG: intervention group
M.I.N.I.: Mini International Neuropsychiatric Interview
PHQ-9: Patient Health Questionnaire-9
PGMM: piecewise growth mixture modeling
RC: reliable change
RCI: reliable change index
SABIC: sample size adjusted BIC.
SD: standard deviation
SF-12: Short-Form Health Survey-12
